# Alcohol Related Changes in Regulation of NMDA Receptor Functions

**DOI:** 10.2174/157015908783769662

**Published:** 2008-03

**Authors:** József Nagy

**Affiliations:** Gedeon Richter Plc., Pharmacological and Drug Safety Research, Laboratory for Molecular Cell Biology, Budapest 10. P.O. Box 27, H-1475 Hungary

**Keywords:** Alcohol dependence, NMDA receptor, subunit expression, post-translation modifications, phosphorylation/ dephosphorylation, compartmentalization.

## Abstract

Long-term alcohol exposure may lead to development of alcohol dependence in consequence of altered neurotransmitter functions. Accumulating evidence suggests that the N-methyl-D-aspartate (NMDA) type of glutamate receptors is a particularly important site of ethanol’s action. Several studies showed that ethanol potently inhibits NMDA receptors (NMDARs) and prolonged ethanol exposition leads to a compensatory “up-regulation” of NMDAR mediated functions. Therefore, alterations in NMDAR function are supposed to contribute to the development of ethanol tolerance, dependence as well as to the acute and late signs of ethanol withdrawal.

A number of publications report alterations in the expression and phosphorylation states of NMDAR subunits, in their interaction with scaffolding proteins or other receptors in consequence of chronic ethanol treatment. Our knowledge on the regulatory processes, which modulate NMDAR functions including factors altering transcription, protein expression and post-translational modifications of NMDAR subunits, as well as those influencing their interactions with different regulatory proteins or other downstream signaling elements are incessantly increasing. The aim of this review is to summarize the complex chain of events supposedly playing a role in the up-regulation of NMDAR functions in consequence of chronic ethanol exposure.

## INTRODUCTION

Alcoholism is a devastating disease defined as a chronic and progressive disorder that includes development of tolerance to alcohol (ethanol), alcohol dependence, i.e. an uncontrolled consumption of alcohol despite the negative consequences, and craving and/or manifestation of withdrawal syndrome when alcohol is removed. Alcoholism ranks as one of the main current threats to the health and safety of people in most Western countries, affecting about 14 million people in the United States with a society cost about $165 billion a year [155]. Long-term and uncontrollable alcohol consumption can cause other disorders that include: antisocial personality disorder, mood and anxiety disorders.

Investigation targeting mechanisms underlying the actions of ethanol in the central nervous system (CNS) has been ongoing for more than a century. Early research on the actions of alcohol focused on the physical properties of alcohols and established the widely appreciated Meyer-Overton relation that describes the direct relationship between the hydrophobicity of an alcohol and its potency for producing intoxication [113, 134]. According to this “lipid theory” of alcohols’ action, ethanol is thought to produce its effects by actions on the membrane lipids bringing about changes in the function of the membrane proteins. Consistent with this hypothesis, the intoxicating potency of aliphatic n-alcohols with up to five carbon atoms is correlated with both their lipid solubility and their membrane lipid disordering potency. However, the potency of aliphatic n-alcohols for producing intoxication reaches a maximum at six to eight carbon atoms and then decreases [2, 54, 138]. The molecular bases of this “cut-off” effect cannot be explained by the “lipid theory”, since both lipid solubility and membrane lipid disordering potency increase exponentially with the increase in the carbon chain length. The cut-off in ethanol’s effect as well as data demonstrating that definite subunit composition and certain amino-acid residues of sensitive receptors are critical for allosteric modulation by ethanol challenged the hypothesis of ethanol’s non-specific membrane effects [3, 88, 114, 178].

Although, the exact mechanism by which ethanol exerts its effect is still a matter of debate, studies over the last decade showed that ethanol, affecting several neurotransmitter systems, differentially modifies processes of neurotransmission in the CNS. Besides altering the release of neurotransmitters like dopamine (DA) - resulting in increased DA levels in the “central reward” pathway -, ethanol alters the function of a number of neurotransmitter receptors (e.g.: γ-amino butyric acid A (GABA_A_), glycine, glutamate, nor-epinephrine, DA, serotonin, acetylcholine) as well as transporters (adenosine, nor-epinephrine, DA, serotonin). Especially the ligand-gated ion channels including those belonging to the brain's major amino acid neurotransmitter systems – the inhibitory GABA and the excitatory glutamate receptors – were shown highly sensitive to the acute effect of ethanol at relevant (<100 mM) concentrations [49, 96, 103]. In the past years, there has been increasing evidence of facts that acute ethanol facilitates GABAergic transmission by enhancing chloride conductance of the GABA_A_ receptors, and inhibits glutamatergic function by decreasing the flow of cations especially through the NMDA activated subclass of glutamate receptors. Furthermore, after chronic ethanol consumption, reduced GABAergic and increased glutamatergic functions have been observed and associated with neurochemical mechanisms underlying the development of alcohol tolerance and dependence. Among glutamate receptors, AMPA and especially NMDA type of receptors were shown to represent the highest affinity targets for ethanol in the CNS [56, 57, 66, 86, 102]. Biochemical, electrophysiological as well as behavioral evidences also supported the conception that ethanol can serve as a potent and selective inhibitor of these receptors [31, 37, 65, 102, 104].

## STRUCTURE AND FUNCTION OF NMDA RECEPTORS

NMDA receptors belong to the family of ionotropic glutamate receptors, and are abundantly expressed in the CNS. The involvement of these receptors in diverse neural functions like excitatory synaptic transmission, synaptic plasticity, and excitotoxicity rests upon their unique features, i.e. their special characteristics like high permeability to Ca^2+ ^ions, the relatively slow activation / deactivation kinetics and their voltage-sensitive blockage by Mg^2+ ^ions. Due to this Mg^2+^ block occurring physiologically at voltages close to the resting membrane potential, glutamate, the native agonist of these receptors can open the ion-channel only if the cell is depolarized, typically upon AMPA/kainate receptor activation. Subsequent to relief of the Mg^2+^ block, NMDARs permit the entry of Ca^2+ ^ions that can activate secondary messenger cascades. By this means, NMDARs act as coincidence perceptive elements, which become active only when electrical and chemical signals are present concurrently [3].

**Fig. (1).Schematic model of NMDA glutamate receptor illustrating multiple regulatory sites.**
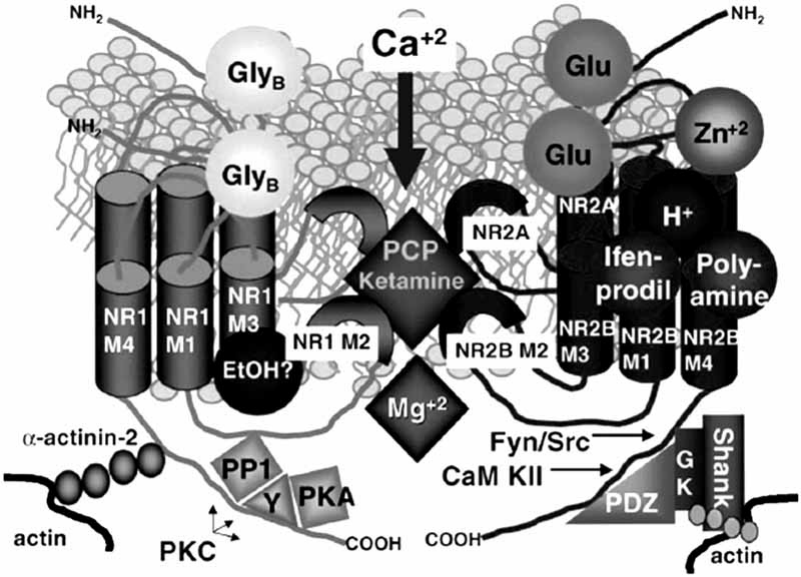
The NMDA glutamate receptor appears to be comprised of 4 subunits, including 2 NR1 subunits and 2 NR2 subunits. This figure illustrates a receptor with an NR2A subunit (in the rear) and an NR2B subunit (in front). Each subunit contains 4 transmembrane domains (TM1–TM4),of which TM2 constitutes a key element of the Ca^2+^ pore (Kupper *et al.*, 1996). The TM3 region of the NR1 subunit, and perhaps other subunits, appears to be associated with ethanol binding (Ronald *et al.*, 2001). The N-terminal cassette does not appear to be a binding site for ethanol (Popp *et al.*, 1998) but does contain critical sites for modulation, including Zn^2+^ and redox agents (NR2A) or protons, polyamines,and ifenprodil (NR2B) (reviewed in the text and in Paoletti *et al.*, 1997, 2000; Choi *et al.*, 2001). The glycine-B (GlyB) sites are located in the extracellular domains. The intracellular cassettes of the NR1 contain the sites at which PKA and PKC phosphorylate and PP1 dephosphorylates this subunit. Yotiao (Y) is an NR1-binding protein. The NR1 subunit also contains a site that attaches this subunit to the cytoskeleton *via* α-actinin-2. The intracellular domain of the NR2 subunits contain phosphorylation sites for the tyrosine kinases (Fyn and Src),CaM kinase II (CaM KII), and PDZ-containing anchoring proteins (PSD-95, chapsyn-110/PSD-93, and SAP-102) that in turn attach NR2 subunits to the cytoskeleton *via* other proteins (GKAP, Shank, and cortactin). Modified from Woodward (2000) and Millan (2002).John H. Krystal, Ismene L. Petrakis, Graeme Mason, Louis Trevisan, D. Cyril D’Souza (2003) N-methyl-D-aspartate glutamate receptors nd alcoholism: reward, dependence, treatment, and vulnerability. *Pharmacol. Ther.,* **99 **, 79-94).

Besides glutamate, NMDARs are sensitive to several other endogenous modulators. For full receptor activation, the co-agonist glycine [112] or D-serine [120] must also bind to the receptor. Endogenous polyamines, spermine and sper-midine also facilitate [95, 150], whereas extracellular protons [169, 175] and Zn^2+^ ions [27] suppress NMDAR activation. In addition, NMDARs interact with various intracellular scaffolding, anchoring, and signaling molecules associated with the postsynaptic density (Fig.**1**). The sensitivity of NMDARs to different ligands, its permeation and blockade by divalent ions, kinetic properties, and interaction with intracellular proteins highly depend on their subunit composition [32, 74].

**Fig. (2).NMDAR subunit diversity.**
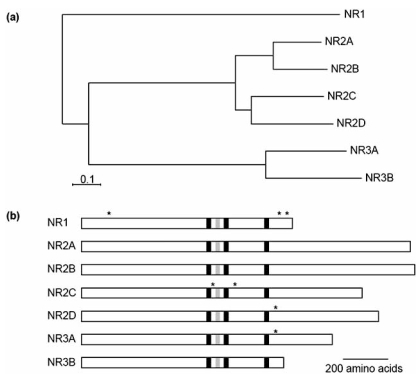
**(a)** Dendrogram of complete amino-acid sequences for rat NMDAR subunits. **(b)** Representation of NMDAR subunit polypeptides.Black boxes indicate transmembrane domains, and grey boxes show the transmembrane TM2 re-entrant loop. Asterisks denote regions at which alternative splicing takes place. This is best characterized for the NR1 subunit, which has three regions of alternative splicing: the amino-terminal N1 cassette (exon 5); and the carboxy-terminal C1 (exon 21) and C2 (exon 22) cassettes. Splicing at these sites can generate eight distinct isoforms (NRI-1a, -1b, -2a, -2b, -3a, -3b, -4a and -4b). Splicing of the NR2C subunit leads to truncated polypeptides ending after TM1 or TM3. The NR2D subunit can be spliced in the carboxyl terminus, producing a 33-amino-acid insert. Likewise, NR3A splicing leads to a 20-aminoacid insert in the carboxyterminal domain. NR2B, NR2C and NR2D also have splice sites in their 5'-untranslated regions but no splice variants have been reported for NR2A. (Stuart Cull-Candy, Stephen Brickley and Mark Farrant (2001) NMDA receptor subunits: diversity, development and disease.*Curr. Opin. Neurobiol.,* 11, 327-335).

**Fig. (3).Potential sites for ligand binding at NMDARs.**
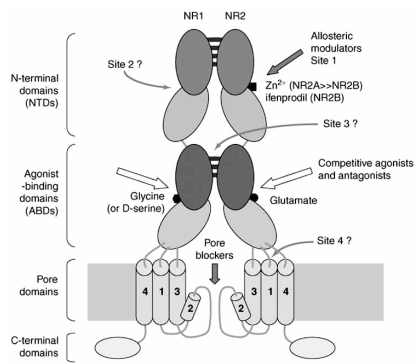
Most NMDAR are believed to assemble as tetramers, associating two NR1 and two NR2 subunits in a ‘dimer of dimers’ quaternary architecture.For clarity, only one of the two NR1/NR2 heterodimers is shown. The extracellular region of each subunit is made up of a tandem of bilobate ‘Venus-flytrap’ domains, the NTD and the ABD. In the extracellular region, the subunits dimerize at the level of the ABDs and probably also at the level of the NTDs. The NR2 ABD binds glutamate, whereas the NR1 ABD binds the co-agonist glycine (or D-serine).White arrows indicate binding sites for competitive agonists and antagonists. Thick orange arrows indicate sites known to bind allosteric modulators such as endogenous zinc (NR2A and NR2B NTDs) or ifenprodil-like compounds (NR2B NTDs), both acting as non-competitive antagonists. The ion-channel domain also forms binding sites for pore blockers such as endogenous Mg^2+^, MK-801, memantine or ketamine,acting as uncompetitive antagonists. Thin orange arrows indicate putative modulatory sites, which can bind either positive or negative allosteric modulators. The only known NMDAR antagonists that display strong subunit selectivity are the NR2 NTD ligands Zn^2+^, which selectively inhibits NR2A-containing receptors at nanomolar concentrations, and ifenprodil-like compounds, which selectively inhibit NR2Bcontaining receptors. (Pierre Paoletti and Jacques Neyton (2007) NMDA receptor subunits: function and pharmacology. *Curr. Opin. Pharmacol.,* 7, 39-47).

NMDARs, like other ion-channel receptors, are multimeric transmembrane proteins, assembled of different types of subunits. Several distinct NMDAR subunits have been identified in neurons (Fig. **2**). The ubiquitously expressed NR1 subunit occurs as eight distinct isoforms because of three independent sites of alternative splicing (N1, C1 and C2 cassettes). Families of four NR2 (A, B, C and D) and two NR3 (A, B) subunits are also identified [35, 67, 118]. Although, the precise subunit composition and stoichiometry of native NMDARs are difficult to determine and largely unknown [92, 141], it is believed that NMDARs exist as tetrameric complexes consisting of at least one NR1 and one NR2 subunits [116-118]. The subunits are most probably arranged as dimer of dimers with an NR1-NR1-NR2-NR2 orientation in the channel [160]. Each subunit has four hydrophobic regions, although only three of them form membrane-spanning domains (TM1, TM3, and TM4). The fourth one (M2) makes a hairpin bend within the membrane and participate in the formation of the ion-channel pore [32] (Fig. **3**).

Two important composition-dependent properties of the NMDARs include their single-channel conductance [21] and their block by extracellular Mg^2+^ ions. Diheteromeric NMDARs composed of NR1/NR2A or NR1/NR2B subunits produce ‘high-conductance’ channel openings with a high sensitivity to Mg^2+^ blockade. On the contrary, receptors containing NR2C or NR2D subunits give rise to ‘low-conductance’ channels with a lower sensitivity to extracellular Mg^2+^ ions. The NR3 subunits are thought to be regulatory in nature since they do not form functional channels with NR1 but can co-assemble with NR1/NR2 complexes [35]. NMDARs containing NR3 subunits form ‘low-conductance’ channels. Insertion of an NR3 subunit into NR1/NR2A complexes show a roughly five-fold reduction in relative Ca^2+^ permeability compared to those composed of merely NR1/NR2A subunits [139].

One of the NR2 subunits, the NR2B type appears to be especially important in respect of several functional properties of the NMDAR. It determines channel’s sensitivity to Mg^2+^ block. Replacement of Trp607 of this subunit to Asp or Ala greatly attenuates while substitution of this amino acid side chain for Leu completely abolishes the ability of Mg^2+^ to block NMDAR function. According to these observations, Williams *et al*. [187] proposed a model in which the M2 loop of the NR2B subunit containing the Trp607 is part of the binding site for Mg^2+^ ions. The presence of an NR2B subunit in the receptor complex affects the binding of the co-agonist glycine as well. Whereas the binding site for glycine is thought to be located on the NR1 subunit [32], a high-affinity site for CGP61594, a glycine site antagonist, was shown exclusively displayed by NR1/NR2B receptors as compared to receptors containing other assemblies of NR2 subunits [68]. One of the unique characteristics of the NR2B subunit is that it binds polyamines. Polyamines appear to facilitate NMDAR function, probably by reducing the inhibitory impact of protons [52]. The NR2B subunit is a highly relevant candidate for mediating the modulatory effects of polyamines, such as spermine and spermidine. In addition, similarly to polyamines, ifenprodil [8], the prototype of the recently discovered NR2B subunit selective NMDAR antagonists (SSNAs) and related compounds – e.g. eliprodil, CP-101,606, Ro25-6981 or RG-1103 [9, 18, 51, 78, 185, 186] – selectively antagonize NMDA responses in those receptors that contain NR2B subunit.

## EFFECTS OF ETHANOL ON AGONIST INDUCED NMDAR ACTIVATION

Ethanol withdrawal is thought to be a physiopathological state associated with increased number and function of NMDARs. Primarily this conception was based on the observations that in ethanol-withdrawn animals, after interruption of the chronic ethanol treatment, the focal application of NMDA was five-fold more potent than in control animals. In addition, the non-competitive NMDAR antagonist dizocilpine (MK-801) or ethanol markedly reduced the NMDA-induced increase in glutamate levels. These results are consistent with the up-regulation of NMDARs by chronic ethanol exposure and supply biochemical evidence for the presence of NMDARs facilitating glutamate release possibly through positive feedback mechanisms [42, 157].

Levels of aspartate, glycine, and N-acetylaspartylgluta-mate in cerebrospinal fluid (CSF) obtained from alcohol dependent patients were all higher than those of the comparison subjects. On the contrary, the concentration of GABA in their CSF was lower. In addition, there were significant correlations between excitatory neurotransmitters and oxidative stress markers, which suggest that the two mechanisms may play an interactive role in neurotoxicity mediated by ethanol withdrawal [177].

Clinical investigations and animal experiments showed that chronic alcohol consumption leads to elevated plasma levels of the NMDAR co-agonist, homocysteine [12, 13, 15]. Homocysteine has a role in biochemical cascades involving overstimulation of NMDARs, oxidative stress, mitochondrial dysfunction, activation of caspases and DNA damage. These mechanisms are thought to be important in the pathogenesis of both excitotoxicity and apoptotic neurotoxicity. Increased levels of homocysteine in chronic alcoholism was also shown to be associated with brain atrophy, therefore high plasma homocysteine levels are suggested as a good predictor of alcohol withdrawal seizures and brain damage giving opportunity for early anticonvulsive and neuroprotective intervention [13, 14, 16].

In an *in vitro* study, we investigated the alterations in the inhibitory activity of different NMDA receptor antagonists due to chronic and repeated ethanol pre-treatment in primary cultures of rat cortical and hippocampal neurons [126]. The inhibitory activity of the channel blocking agent MK-801 or the glycine-site selective antagonist 5,7-DCK was unchanged in primary cortical cultures after 3 days of repeated ethanol (100 mM) pre-treatment. This is in agreement with the previous findings of al-Qatari *et al*. [6], who observed no changes in the effect of the competitive NMDA receptor antagonist D-APV on NMDA induced calcium entry in ethanol pre-treated neocortical cultures. On the contrary, we found that NR2B subunit selective antagonists – ifenprodil, CP101,606 and CI1041 – as well as ethanol itself reduced NMDA induced calcium responses more potently in the ethanol pre-treated than in control cultures. The maximal inhibitory effects of these compounds significantly increased, and their IC_50_ values slightly, although insignificantly decreased due to ethanol pre-treatment. Similarly, the effectiveness of ifenprodil was shown to be increased in cortical cultures after 4 days of 100 mM ethanol pre-treatment [17], and supersensitivity to ethanol was also observed in pontine slice preparations from ethanol treated (3 g/kg daily for 14 days, i.p.) rats 24 h after the last ethanol dose [140]. NMDA evoked rise in cytosolic calcium level as well as NMDA induced excitotoxicity were found to be potentiated in ethanol-treated primary cortical and hippocampal cultures, too [1, 125, 159, 163]. Furthermore, after several days of ethanol (100 mM) pre-treatment followed by 24-hour withdrawal, increased cell death was observed in primary cultures of rat cortical neurons [123] and in rat entorhinal/hippocampal slice cultures [28]. In this later model the cytotoxic effect of ethanol withdrawal was enhanced by the NMDA receptor co-agonist spermine [142]. Taken together, these data support the conception that enhanced efficiency of NR2B subunit selective antagonists supposedly in consequence of increased expression of the NR2B subunit containing receptor complexes underlies the augmentation of NMDAR functions.

Besides the above mentioned NR2B subunit selective antagonists, the novel abstinence-promoting drug acamprosate (calcium acetylhomotaurinate) used in the treatment of alcohol dependence was also shown to effectively inhibit the cytotoxic effect of ethanol withdrawal in ethanol pre-treated primary cortical cultures [127]. Even though, it did not inhibit the NMDA-evoked cytoplasmic calcium elevation in neurons up to 300 μM, it showed a 75% reduction in withdrawal-evoked LDH release at the same concentration. Although the precise mechanism of action of acamprosate is not clearly known, there is an evidence for an interaction between acamprosate and the NMDARs based on electrophysiological as well as radioligand binding studies. Initial electrophysiological studies showed that acamprosate depressed excitatory postsynaptic potentials mediated by glutamic acid and selectively inhibited direct activation of these same neurons by NMDA in rat cortical slices *in vitro *[202]. However, later studies described potentiation of NMDA responses in hippocampal slices [107]. In addition, Popp and Lovinger [141] showed that acamprosate acts at the polyamine site on the NMDAR and inhibits potentiation of NMDAR activation by the polyamine site ligand spermine. According to these observations acamprosate seems to interact with a regulatory site on the NMDAR, such that the magnitude and direction of the effect of acamprosate is determined by the resting level of NMDAR activity. Naassila *et al*. [122] described a low-affinity but saturable binding site for [^3^H]-acamprosate on rat brain membranes. The binding of [^3^H]-acamprosate could be displaced by spermidine and other polyamines and they described also the displacement of [^14^C]-spermidine binding by acamprosate at similar concentrations. In another study, al Qatari *et al*. [5] reported that under conditions of low NMDA receptor activation, acamprosate potentiated [^3^H]-dizolcipine binding, whereas, at high levels of receptor activation, binding was inhibited. Interestingly, the potentiating effect of acamprosate on [^3^H]-dizolcipine binding was lost in membranes prepared from rats made dependent on alcohol. This finding was explained by a change in the subunit composition of the NMDA receptor as a result of chronic alcohol exposure. Taken together, the electrophysiological and radioligand binding studies discussed previously are compatible with the hypothesis that acamprosate has a partial agonist activity at an allosteric regulatory site, possibly the polyamine site, on the NMDAR. The interaction of acamprosate with this site is activity-dependent, it means that at low levels of receptor activation, acamprosate would substitute for polyamines and facilitate NMDA receptor activation and at high levels of receptor activation, acamprosate would have an antagonist effect, attenuating receptor activation [36].

Neurosteroids are also important modulators of NMDARs. In various brain areas, neurosteroid concentrations change in response to alterations of particular physiological conditions, and during behavior associated with stress, sexual activity or aggressiveness [29, 136]. In all probability, neurosteroids influence cerebral activity by directly affecting the function of membrane receptors. Neurosteroids known to interact with GABA_A_ and NMDA receptors include: 3β-hydroxy-5-pregnen-20-one-3-sulfate (pregnenolone sulfate) (PS); dehydroepiandrosterone sulfate (DHEAS); 3α-hydroxy-5α-pregnan-20-one (3α5α allopregnanolone); 3α-hydroxy-5β-pregnan-20-one (3α5β pregnanolone) and 3α-hydroxy-5β-pregnan-20-one sulfate (3α5βS pregnanolone sulfate) [91, 108, 135]. Previously it was suggested that estrogen and PS might directly modulate NMDA receptors increasing both the frequency of opening and the mean open time of the ion-channel [19, 191]. Furthermore, acute administration of alcohol was shown to increase the concentrations of the neurosteroids, preg-nenolone and allopregnanolone [142]. Conversely, during EtOH withdrawal decreased endogenous allopregnanolone levels were found in seizure-prone animals [45, 75]. Costa *et al*. showed that prenatal ethanol exposure can modify the allosteric modulation of NMDARs by neurosteroids. Primary cultures of hippocampal neurons were prepared from the neonatal offsprings of dams exposed to an ethanol-containing liquid diet that results in blood ethanol levels near the intoxication limit, and NMDA receptor function was assessed by patch clamp electrophysiological techniques after 6-7 days in culture in ethanol-free media. Whereas, they did not detect any change in hippocampal NMDA receptor function at either the whole-cell or single-channel levels, they observed alterations in the actions of the neurosteroids pregnenolone sulfate and pregnenolone hemisuccinate without any change in NMDAR subunit expression [30].

## EFFECTS OF ETHANOL ON NMDAR SUBUNIT EXPRESSION 

Several *in vivo* and *in vitro* studies showed that chronic ethanol treatment leads to an increased density of NMDARs leading to facilitation of receptor functions [48, 50, 69, 70, 71, 122, 189]. For instance, binding of the channel blocking agent [^3^H]MK-801 to brain membranes from rats exposed to severe alcohol intoxication was increased by 49% after the last alcohol dose compared with the control group [62]. However, no chronic ethanol exposition-related up-regulation of NMDAR expression was found in other studies [44, 158].

Consistently with the recently emerged view, increased NMDAR function to ethanol treatment is presumably due to a differential up-regulation of the various NMDAR subunits. Notwithstanding, there is a disagreement in respect of the expression of different NMDAR subunits and NR1 splice variant forms. Some authors reported no changes in subunit expression at all [23], while others found changes in the expression of multiple types of subunits or solely in the expression of the NR1 [46] or the NR2A [34] subunit in consequence of long-term ethanol exposure. Numerous* in vitro* studies showed increased NR2B subunit mRNA levels, with no change in NR1 and/or NR2A subunit transcription in cultured cortical neurons following chronic ethanol administration [61, 70, 119]. For example, although the NMDAR function was enhanced in the lateral/basolateral amygdala and the increase in the NMDAR current density was associated with an increase in ifenprodil inhibition after chronic ethanol ingestion, quantitative real-time reverse transcription-poly-merase chain reaction (qRT-PCR) measurements demonstrated no changes in NR2 or NR3 subunit transcription [46, 188]. In another study, there were not significant changes observed in any of the NMDA subunit mRNA and protein expressions suggesting that the functional NMDA receptor adaptations are likely to be mediated by post-translational events [90].

On the contrary, there are several reports in which the levels of NR2B as well as NR2A and NR1 subunit proteins were found increased in the cortex and hippocampus of ethanol treated rats or mice [47, 48, 64, 76, 89, 127, 176]. Similarly, exposure to 50 mM ethanol caused an increase in the levels of NR1, NR2A and NR2B subunits in cultures of rat hippocampal neurons. In addition, the NMDAR antagonist compound memantine completely blocked the ethanol-induced up-regulation of these subunits [110]. Also, after chronic ethanol treatment (CET), when rats were exposed to continuous ethanol vapors for at least 2 weeks, the mRNA levels of NR1 and NR2B NMDAR subunits were significantly increased compared to control. Western blot analysis revealed a significant increase in NR1 and NR2B NMDAR subunit proteins occurred in central nucleus of the amygdala from chronically ethanol treated rats that reversed after 2 weeks of withdrawal [149]. In addition, higher NR2B subunit level was found in cultured cerebellar granule cells, resulting in a delay in the 'developmental switch' of the NR2B subunit for NR2A [164].

In accordance with the above observations, the maximal inhibitory effect of ethanol as well as some NR2B subunit selective NMDAR antagonists on NMDA evoked cytosolic calcium elevations was significantly increased in ethanol pre-treated primary cultures of cortical as well as hippocampal neurons obtained from rats. On the other hand, the efficiency of the non-subunit selective NMDAR antagonists, the channel blocker MK-801 and the glycine site specific 5,7-DCK was not changed. Correspondingly, increased expression of the NR2B subunit protein could be detected by applying a flow cytometry-based immunocytochemical method. This quantitative analysis showed that whereas the non splice variant specific immuno-staining of the NR1 and the subunit specific immuno-staining of the NR2A, NR2C, and NR2D subunits were not changed, the NR2B specific immuno-labeling was increased in a subpopulation of cells in ethanol pre-treated compared to control cultures. In further studies, when the expression of the different NR1 splice variants was investigated, in addition to the NR2B subunits, the expression of the C1 (exon 21) and C2’ (exon 22) cassette containing splice variants was found to be increased in ethanol pre-treated hippocampal cultures [126].

The molecular mechanisms underlying these changes in subunit expression, i.e. the effect of ethanol on the regulation of subunit transcription is still an open question. First results concerning the regulation of NMDAR subunit composition showed that the methylation status of the NR2B gene was altered following chronic ethanol treatment in mouse cortical neurons [147]. The authors found that demethylation of this gene could be responsible for the up-regulation of the NR2B subunit expression. Although methylation in CpG islands in the regions (5843-6276) and (6477-6763) of the NR2B gene was not influenced by acute ethanol treatment, chronic ethanol promoted demethylation in this area suggesting that demethylation is likely to contribute to the increased expression of the NR2B gene. In another experiment, it was shown that a 467 base-pair DNA fragment of the NR2B promoter containing the cAMP responsive element (CRE) sequence cloned into a luciferase reporter vector exhibited high activity in transient co-transfection assay of mouse cortical neurons, and ethanol treatment increased this activity. Introducing site-directed mutation in the CRE sequence significantly reduced the reporter activity relative to the wild-type construct, and it also abolished the stimulatory effect of ethanol. These results indicate that CRE binding protein (CREB) is probably involved in the mediation of ethanol-induced up-regulation of the NR2B gene [146].

Another study addressed the role of the neuron-restrictive silencer factor (NRSF) - a transcriptional repressor of multiple neuronal genes - in NR2B promoter activity and the molecular mechanisms of ethanol-induced NR2B up-regulation in fetal cortical neurons [143]. Neuron-restrictive silencer element (NRSE) motifs located between base pair -1407 and -2741 repressed transcription of the NR2B gene. Treatment of cultured cortical neurons with 100 mM ethanol for 5 days caused a significant decrease in the NRSF mRNA and protein levels, NRSF/NRSE binding activity, and an increase in the promoter activity. Therefore, it was suggested that NRSF acting as a negative regulator of NR2B expression may contribute to the ethanol-induced up-regulation of the NR2B gene in fetal cortical neurons. One more factor, the tissue-plasminogen activator (tPA), a protease implicated in neuronal plasticity and seizures, is also thought to interact with NR2B-containing NMDA receptors and suggested to play a role in up-regulation of the NR2B subunit in response to ethanol. tPA is induced in the limbic system by chronic ethanol consumption, temporally coinciding with up-regulation of NMDA receptors. Furthermore, tPA-deficient mice have reduced NR2B, extracellular signal-regulated kinase 1/2 phosphorylation and seizures after ethanol withdrawal (EW), and tPA-mediated facilitation of EW seizures was abolished by the NR2B-specific NMDA antagonist ifenprodil. These results indicate that tPA may mediate the development of physical dependence on ethanol by regulating NR2B-containing NMDA receptors [137].

Regarding the effect of ethanol on NR1 subunit expression, Kumari and co-workers observed that chronic ethanol exposure of fetal cortical neurons selectively increased the expression of NR1 splice variants lacking exon 5 and exon 22 [89]. Also the half-life of NR1 mRNAs was increased in neurons chronically exposed to ethanol. However, when new protein synthesis was inhibited, the NR1 mRNA half-life returned to control values, strongly suggesting that ethanol induced the synthesis of certain protein(s) that may regulate the decay of NR1 mRNA. In recent years, it has become apparent that regulation of mRNA stability is an important aspect of regulation of gene expression. Changes in mRNA stability can be accomplished by interaction between cis-acting sequences in the 3' untranslated region (3'UTR) of mRNAs and trans-acting proteins. Such interactions may protect RNAs from degradation by ribonucleases, thereby increasing the half-life of mRNAs. Another possible mechanism regulating mRNA half-life is RNA interference. RNA interference (RNAi) is a process whereby small non-coding RNAs (microRNAs) silence specific genes. So far, there is no data available regarding the regulation of NMDAR expression by microRNAs.

## EFFECTS OF ETHANOL ON PROTEIN-PROTEIN INTERACTIONS

### Altered Post-Translational Modifications of NMDARs 

The most frequent post-translational modification of a newly synthesized protein is phosphorylation, i.e. the addition of a phosphate group to serine, threonine, or tyrosine residues of a protein substrate. This reaction is achieved *via* activation of different protein kinases classified into three groups: serine/threonine kinases, tyrosine kinases, and dual-specificity kinases, which are able to phosphorylate all three residues. The opposite reaction, i.e. dephosphorylation, in which a phosphate group is removed from a protein, is performed by either serine/threonine or tyrosine phosphatases. Whereas receptor kinases and phosphatases are integral parts of the plasma membrane, the non-receptor kinases and phosphatases reside intracellularly. Kinases and phosphatases are specifically activated by signals that are usually transduced from outside to inside the cell. For example, activation of membrane receptors results in the generation of second messengers which can in turn initiate signaling cascades including the activation of kinases and/or phosphatases. The phosphorylation state of a protein therefore reflects the balance between phosphorylation and dephosphorylation events [155].

Phosphorylation plays a central role in regulation of NMDAR function. It results in the enhancement of channel function because application of tyrosine kinase inhibitors inhibits whereas that of tyrosine kinases (e.g. Src kinase) enhances NMDAR-mediated currents [25, 182, 200, 201]. Many NMDAR functions have been linked to tyrosine phosphorylation of the NR2 subunits of the NMDAR. NR2A and NR2B are phosphorylated on serine and tyrosine residues, and the alternatively spliced isoforms of the NR1 subunit is phosphorylated on serine residues. The highly homologous tyrosine kinases Fyn and Src have been implicated thus far in phosphorylating the NR2 subunits on tyrosine residues on sites depicted in Fig. (**4**) [82, 128, 167, 170, 173, 196]. It is unclear, however, whether both kinases phosphorylate both subunits under physiological conditions, or which sites are phosphorylated by which kinase, and whether different signaling pathways activate the two kinases. Both Fyn and Src have been implicated in playing an important role in development of long term potentiation (LTP). NR2B phosphorylation is enhanced after the induction of (LTP) in the CA1 region of the hippocampus [83, 128], whereas inhibitors of the Src tyrosine kinases prevent the appearance of LTP [105]. In addition, LTP is blunted in mice in which the fyn gene was deleted [56]. Tyrosine phosphorylation of NR2 subunits has also been found to regulate the trafficking of these subunits in striatal neurons [40]. In addition, tyrosine phosphorylation of the NR2 subunits was observed in taste aversion learning [156], during transient global ischemia [168], and after acute injection of hypnotic doses of ethanol [115, 197].

At least in part, the inhibitory effect of ethanol on NMDAR function could be due to a change in the phosphorylation state of NMDARs. The role of phosphorylation to modulate the sensitivity of NMDARs for ethanol has begun to be investigated by Miyakawa *et al*. [115, 192]. They showed that mice deficient in the non-receptor Fyn tyrosine kinase were more sensitive to the hypnotic effects of alcohol and that this effect was correlated with a lack of phosphorylation of the NR2B subunit. This suggested that Fyn kinase may regulate ethanol sensitivity of NR2B containing NMDA receptors in wild-type animals. Anders *et al*. [7] examined this question directly by using a recombinant expression system and found no evidence to suggest that Fyn kinase affects the ethanol sensitivity of NR1/NR2B receptors. Interestingly, in another study, the inhibitory effect of a high concentration of ethanol (100 mM) on NR1/NR2A currents was reduced by Fyn kinase. In order to investigate the effect of ethanol on the phosphorylation state of NR1 and NR2 subunits, NMDAR complexes were immunoprecipitated from rat cortical slices pre-exposed to ethanol. Acute ethanol (100 and 200 mM) significantly decreased the tyrosine phosphorylation of NR2 subunits (Tyr-NR2). Treatment with a tyrosine phosphatase inhibitor reduced the inhibition of Tyr-NR2 phosphorylation caused by ethanol. This suggests an involvement of tyrosine phosphatases in ethanol-induced inhibition of Tyr-NR2 phosphorylation [43]. Correspondingly, Alvestad *et al*. found that tyrosine phosphorylation of both NR2A and NR2B subunits was significantly reduced following *in situ *exposure of hippocampal slices to 100 mM ethanol.

**Fig. (4).Phosphorylation sites of NMDAR subunits.**
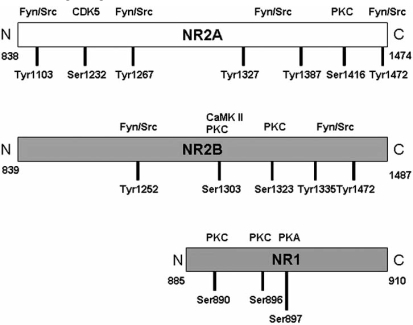
The N-methyl-D-aspartate (NMDA) receptor subunits are phosphorylated on serine and tyrosine residues. Depicted are the amino acid sequences of the cytoplasmic tails of rat NR1, NR2A, and NR2B subunits. The N-terminus faces the membrane, and the C terminus is facing inward. The kinase is depicted on the top of each rectangle, and the amino acid residue is depicted at the bottom. PKC, protein kinase C;PKA, protein kinase A. (Adapted from: Ron, D. (2004) Signaling Cascades Regulating NMDA Receptor Sensitivity to Ethanol. *Neuroscientist,* **10**(4), 325-336.)

Serine/threonine kinases like cAMP-dependent protein kinase A (PKA), protein kinase C (PKC), calcium-/calmo-dulin-dependent protein kinase II (CaM kinase II) and cyclin-dependent protein kinase 5 (CDK5) also phosphorylate the NMDAR subunits at different sites (Fig. **4**). PKA phosphorylates the NR1 subunit on residue 897 [93, 174], and this phosphorylation has recently been found to be essential for dopamine D1 receptor mediated phosphorylation of the transcription factor CREB [37]. In addition, activation of postsynaptic PKA has been reported to enhance channel activity [144] and to be important for the expression of LTP in the CA1 region of the hippocampus [39]. In a study of Xu and Woodward [194], the inhibitory effect of ethanol on ion currents through recombinant NMDA receptors was determined under conditions designed either to enhance or inhibit the activity of PKA. This included treatment of cells with cAMP analogs, inclusion of phosphatase inhibitors or purified PKA in the pipette filling solution, co-expression of catalytically active PKA, expression of the NR1 PKA-site phosphorylation site mimic (S897D) or by co-expression of the PKA scaffolding protein yotiao or the dopamine D1 receptor. The degree of ethanol inhibition was not affected or was slightly enhanced under conditions designed to enhance PKA activity. Similarly, alteration in ethanol inhibition of NR1/NR2A and NR1/NR2B receptors was not observed when PKA effects were suppressed by co-expression of either a PKI inhibitory peptide or the phosphorylation-defi-cient NR1 mutants (S897A, S896A, S896A/S897A). In addition, ethanol inhibition of NMDA-induced currents in cultured cortical or hippocampal neurons was not affected by modulators of PKA. However, in another study, rat cortical slices pre-exposed to ethanol (100 and 200 mM) exhibited a significant increase in the phosphorylation of NR1 by PKA at serine 897 (Ser897-NR1), which was blocked by a PKA inhibitor. Moreover, 200 mM ethanol produced a significant increase in PKA activity [43]. In spite of these contradictory results and taking into account that mice with genetic disruptions in cAMP/PKA signaling exhibit increased ethanol sensitivity and do not develop tolerance to the sedative effect of ethanol [171, 180], PKA may play a substantial role in increased NMDAR activity induced by chronic ethanol treatment.

Another kinase that specifically phosphorylates the NR subunits is PKC. PKC phosphorylates serine residues 890 and 896 on the C1 cassette of the NR1 subunit (Fig. **4**) [93, 174]. Phosphorylation of the C1 cassette regulates the export of the NR1 subunit from the endoplasmic reticulum and the corresponding forward trafficking of this subunit to the synapse [156]. Furthermore, PKC phosphorylation of the NR2A subunit on serine 1416 reduces CaM kinase II binding to NR2A [53], and phosphorylation of the NR2B subunit on residues serine 1303 and 1323 has been implicated in the enhancement of channel function [98]. Finally, PKC activation indirectly induces the tyrosine phosphorylation of NR2A and NR2B subunits [106, 59] *via* the activation of the calcium-dependent tyrosine kinase Pyk2/Cak2β [72]. Systemic ethanol administration alters PKC activity in rat brain. PKCε expression was increased in the cytosol and decreased in the membrane fraction of cerebral cortex. In contrast, PKCγ in the membrane fraction as well as in the cytosol was decreased. PKCβ expression was increased in the membrane fraction both at 10 and 60 min post-ethanol administration, whereas cytosolic levels were unchanged. Phosphorylation of NMDA receptor NR1 subunit was increased 60 min following ethanol administration. These findings suggest that acute ethanol administration alters PKC synthesis and translocation in an isoform and brain region specific manner that leads to alterations in serine phosphorylation of the receptor. Furthermore, chronic ethanol pre-treatment of rats (6% v/v ethanol in liquid diet for 7 days followed by 7.5% v/v ethanol in liquid diet for further 7 days) prevented ethanol-induced alterations in PKC expression in the membrane fraction, where PKC interacts with ethanol-responsive ion channels [87]. Pre-treatment with the γ-isozyme-specific peptide PKC inhibitor, gammaV5-3, blocked ethanol-induced translocation and also blocked withdrawal hyper-responsiveness in rat spinal cord slices showing that PKCγ mediates ethanol withdrawal hyper-responsiveness in spinal motor neurons [97]. According to Sultana *et al*., PKC activity was increased in both the membrane and the cytosolic fraction of cerebellar cells taken from alcohol-treated rats (6 g/kg ethanol per day for 60 days), whereas PKC activity in cerebral cortex was found to be decreased in the membrane fraction with no appreciable changes in the cytosolic fraction. According to an *in vitro* phosphorylation study, the NR2A subunit was hypophosphorylated in cerebral cortex and cerebellum of ethanol-treated rats [166]. In addition, ethanol did not affect the phosphorylation of the NR1 subunit by PKC at serine 896 in rat cortical slices pre-exposed to 100 and 200 mM ethanol [43].

CaM kinase II also phosphorylates NR2B by directly interacting with the subunit [11], and phosphorylating its serine 1303 residue, a site shared with PKC [133, 155]. The activation of this kinase is directly linked to the activation of the NMDAR channel and the subsequent entry of calcium ions [11, 94]. It is unclear, however, whether CaM kinase II and PKC phosphorylate the same site *in vivo* and whether phosphorylation of this site leads to the same or different functional consequences. 

Finally, CDK5 has recently been shown to be activated in forebrain ischemia, resulting in the phosphorylation of NR2A on serine 1232, and consequently leading to CA1 hippocampal neuronal cell death [181]. Effect of ethanol on the activity of this kinase is not yet published.

As mentioned above, the phosphorylation state of a protein reflects the balance between the activity of kinases and phosphatases, and thus phosphatases also play a critical role in regulating NMDAR activity. The serine and threonine phosphatases, calcineurin (also known as PP2B) and protein phosphatase 1 (PP1), are highly important for the regulation of the phosphorylation state and thus functions of NMDARs [60]. Calcineurin requires binding of calcium/calmodulin for its activation and in turn regulates the activity of PP1 *via* dopamine and cAMP-regulated phosphoprotein with molecular weight of 32 kDa (DARPP-32). DARPP-32 is expressed predominantly in the medium spiny dopaminergic neurons of the neostriatum, and it is an important regulator of numerous functions including the regulation of the NMDAR. The activity of DARPP-32 depends on its phosphorylation state. Activation of the cAMP/PKA pathway leads to DARPP-32 phosphorylation on serine 34, and upon phosphorylation, DARPP-32 potently inhibits the phosphatase PP1 [58]. Activation of the cAMP/ PKA pathway also leads to the phosphorylation of NR1 by PKA, and the phosphorylation contributes to the enhancement of channel activity, whereas PP1 dephosphorylates the subunit. This NR1 phosphorylation, together with the inhibition of PP1 by phospho-DARPP-32, keeps the NR1 subunit in a phosphorylated state. Activation of the NMDAR and the accompanying increase in calcium result in the activation of calcineurin, which dephosphorylates both the NR1 subunit and DARPP-32 and leads to a reduction in channel activity [58]. Recently it was reported that this DARPP-32/PP1 cascade is an important regulatory mechanism for neostriatal NMDARs in the presence of ethanol [109]. Ethanol, like other drugs of abuse, increases dopamine release in the neostriatum [84]. Maldve and colleagues found that the activation of the cAMP/PKA pathway *via* the dopamine D1 receptor (D1R) leads to both phosphorylation of NR1 and DARPP-32. The combination of NMDAR phosphorylation and the inhibition of PP1 results in the reduction of the inhibitory action of ethanol on the NMDAR. Interestingly, ethanol itself (*via* a mechanism that has not yet been identified) mediates the phosphorylation of DARPP-32, contributing to the enhancement of NMDAR channel activities. Importantly, this disinhibition of NMDAR channel activity, *via* ethanol and the release of dopamine, is likely to be an important factor in NMDAR-dependent LTP, a process considered important for long-term neuroadaptations to ethanol [109]. In addition, Edwards and co-workers reported that although *in vivo* administration of a dopamine D1R agonist resulted in an increase in the phosphorylation state of both NR1 and DARPP-32, treatment with dopamine D2 receptor (D2R) agonist (which results in an inhibition of the cAMP/PKA pathway) significantly reduced the activities of the D1R agonist when both drugs were co-administered. Ethanol, however, reversed the inhibitory effect of D2R activation, suggesting that ethanol in the neostriatum is synergizing with dopamine D1R to increase NR1 and DARPP-32 phosphorylation, and by doing so, ethanol is masking the effects mediated by D2Rs [41]. Last, the contribution of DARPP-32 to ethanol-mediated behaviors was tested using the DARPP-32 deletion mutant mice (DARPP-32^-/-^) [148]. DARPP-32^-/-^ mice consume less ethanol compared to the wild-type control mice and are less responsive to the rewarding actions of ethanol as measured by the conditioned place preference paradigm, further implicating DARPP-32 as an important specific regulator of ethanol’s behavioral effects.

### Altered Regulation of NMDAR Functions by Other Receptors

In chronically cannulated rats it was found that injection of the group I mGluR agonist 3,5-dihydroxyphenylglycine (DHPG) into the caudate putamen significantly increased phosphorylation of the two serine residues (serine 896 and serine 897) on the intracellular C-terminus of the NR1 subunit. The increase in NR1 phosphorylation was dose-dependent and DHPG had no effect on basal levels of NR1 proteins. Intrastriatal infusion of the group I mGluR anta-gonist N-phenyl-7-(hydroxyimino)cyclopropa[b]chromen-1a-carboxamide (PHCCC) significantly attenuated the DHPG-stimulated NR1 phosphorylation. Pretreatment with the mGluR5 antagonist 2-methyl-6-(phenylethynyl) pyridine hydrochloride (MPEP) also produced the same effect. These data suggest that group I mGluRs, likely mGluR5 receptors, possess the ability to up-regulate protein phosphorylation of NMDA receptor NR1 subunits in striatal neurons *in vivo* [26].

Recently, evidence has emerged that acamprosate, a novel abstinence-promoting agent may interact with excitatory glutamatergic neurotransmission in general and as an antagonist of the metabotropic glutamate receptor subtype 5 (mGluR5) in particular. These findings provide, for the first time, a satisfactory, unifying hypothesis that can bring together and explain the diverse neurochemical effects of acamprosate [36]. Acamprosate has been shown to block the changes in phosphorylation of the NMDAR following mGluR5 receptor activation. The basal phosphorylation state of the NMDAR is likely to be different in alcohol-naive neurons than in neurons that have been chronically exposed to ethanol because of various adaptive changes in the NMDAR. The indirect action of acamprosate on NMDAR function may differ according to the phosphorylation state of the NMDAR and this may therefore provide an explanation for the selective effect of acamprosate on behaviors related to excessive chronic alcohol consumption compared with those related to acute or occasional alcohol exposure. The second functionally important effect of acamprosate relates to an action at mGluR5 receptors that occurs at the presynaptic level. Glutamate released from glutamatergic nerve terminals activates mGluR5 receptors on the synaptic bouton. The subsequent activation of phospholipase C and release of intracellular second messengers mobilize intracellular calcium and thus potentiates transmitter release [165, 172]. This process thus provides a positive feedback control over glutamate release. Acamprosate may block this positive feedback loop and thereby attenuate large surges in glutamate release that occur when the CNS is hyper-excitable, for example following alcohol withdrawal.

It was also demonstrated that cerebellar adenosine modulates ethanol-induced motor incoordination *via* A(1) subtype of adenosine receptors. The adenosine A(1)-selective agonist N(6)-cyclohexyladenosine and antagonist 8-cyclopentyl-1,3-dipropylxanthine functionally opposed the attenuation by glutamate and NMDA and the accentuation by L-glutamic acid diethyl-ester, AP-5, and (+)-MK-801, respectively, of ethanol-induced motor incoordination. These results suggest a functional antagonism between glutamate NMDA and adenosine A(1) receptors exhibiting a co-modulation of ethanol-induced motor incoordination within the cerebellum [33].

Current responses to NMDA in layer V pyramidal neurons of the rat prefrontal cortex were found to be potentiated by the P2 purine receptor agonists, adenosine 5'-triphosphate (ATP) and uridine 5'-triphosphate (UTP). It was suggested that exogenous and probably also endogenous ATP releases vesicular glutamate from astrocytes *via* P2Y4 receptor activation. This glutamate then stimulates group I metabotropic glutamate receptors (mGluRs) of layer V pyramidal neurons and *via* the Gq - phospholipase C - inositol 1,4,5-trisphos-phate - Ca^2+^ - CAMKII transduction pathway facilitates NMDA receptor currents [189].

### Altered Compartmentalization of NMDAR Subunits

In response to prolonged ethanol exposure, neuroadaptive changes in NMDARs may occur also through processes that regulate the synaptic targeting and localization of these receptors [155]. Carpenter-Hyland *et al.* [22], who used confocal imaging and electrophysiology procedures to assess the effects of prolonged ethanol exposure on NMDA receptor trafficking in cultures of hippocampal neurons found an increase in the dendritic clustering of NMDARs in neurons exposed to 50 - 100 mM ethanol for 4 days. This increase was prevented by addition of the PKA inhibitor KT-5720 or by co-exposure to a low concentration of NMDA and was reversed when ethanol was removed from the cultures. Quite the contrary, i.e. no changes were observed in the synaptic content or density of AMPA receptors after ethanol exposure. Electrophysiological measurements on ethanol-treated neurons revealed a similar enhancement in synaptic NMDA currents with no change in AMPA-mediated events. After blocking synaptic NMDA receptors by MK801 trapping, whole-cell responses to NMDA were not different between control and ethanol-treated neurons. 

NMDARs are important components of the postsynaptic density (PSD) structure within postsynaptic membranes of glutamatergic neurons built up of adhesion molecules and numbers of scaffolding/anchoring proteins [79, 80, 162]. Scaffolding proteins are essential for forming connections between trans-membrane receptors with cytoskeleton, for the assembly of kinases and phosphatases in close proximity with receptor molecules (e.g. with NMDARs), and thus for the regulation of activity of signaling proteins within the PSD [131, 73, 179]. More than 80 proteins have been identified within the PSD complex [73, 179]. NMDARs are linked to the cytoskeleton *via* the cytoskeleton-associated proteins α-actinin-2 and spectrin, which interact with both NR2B and NR1 subunits [193, 183]. In the presence of calcium, calmo-dulin competes with and prevents α-actinin-2 from binding to NR1, presumably leading to NMDAR rundown and to redistribution of the channel (Fig. **1**) [193].

Another major scaffolding protein in the PSD is PSD-95 [55]. PSD95, as well as its homologous proteins PSD93, SAP97, and SAP102, directly interacts with NR2A and NR2B *via* its first two PDZ domains. Association of PSD95 with NR2A and/or NR2B subunits is thought to be important for the compartmentalization of the NMDAR complex within the PSD, for channel clustering, and for the recruitment of signaling proteins in close proximity to the NMDAR complexes [10, 85, 129]. However, in a recent study Lim *et al.* [100] using peptides that disrupt the PSD95/NMDAR interaction reported only moderate inhibition of clustering of the receptor. They have not found any changes in NMDAR-mediated currents either, suggesting that the interaction is not crucial for the activity of the channel.

Another anchoring protein important for the regulation of the phosphorylation state and function of the NMDARs is yotiao. Yotiao is a 230-kDa protein recognized by a two-hybrid screen for NR1-interacting proteins [101]. Yotiao was also identified as a PKA-anchoring protein that associates with PP1 [184]. Under resting conditions, interacting with NR1 and active PP1, it ensures the phosphatase to keep NR1 in a dephosphorylated state. However, upon activation of the cAMP/PKA signaling pathway, the catalytic subunit of PKA phosphorylates NR1 to overcome the activity of the phosphatase, leading to PKA-mediated enhancement of channel activity.

A further scaffolding protein that associates with NMDARs is RACK1. RACK1 is a 36-kDa ubiquitously expressed protein that is highly expressed in the brain. RACK1 was originally cloned and identified as a protein kinase C (PKC) anchoring protein [151]. Lately, several further proteins have been identified which interact with RACK1 [111, 155], including signaling proteins such as the phosphodiesterase PDE4D5 [199], the tyrosine phosphatase PTPμ [121], kinases such as Fyn [195], and intracellular tails of different receptors such as β-integrin [99], GABA_A_ [20], the insulin-like growth factor receptor [81], and the NMDAR [195]. The interaction of RACK1 with proteins allows the compartmentalization of signaling proteins and the regulation of diverse cellular activities [111]. A unique feature of RACK1 is its ability to translocate to different intracellular compartments upon activation of specific signal transduction cascades. For example, activation of the PKC signaling path-way induces RACK1 to translocate together with βIIPKC to its substrate site [151, 152, 153], whereas activation of the PKA pathway induces RACK1 translocation to the nucleus [154, 63, 196]. Nuclear RACK1 in turn alters the expression of several genes [63, 196]. More recently, RACK1 has been found to be an important regulator of ribosome assembly and activation *via* its interaction with βIIPKC [24].

RACK1 is also an important regulator of the phosphorylation state and function of the NMDARs [195, 196]. The association between RACK1 and the NMDAR is subunit specific. Only NR2B, but not NR2A or NR1, binds RACK1 [195]. NR2B is a substrate of Fyn kinase, and RACK1 simultaneously binds both proteins. RACK1 prevents the ability of the kinase to phosphorylate the channel, leading to the inhibition of NMDAR-mediated excitatory postsynaptic potentials [195]. The release of RACK1 from the NMDAR complex *via* activation of the cAMP/PKA pathway allows Fyn to phosphorylate NR2B specifically, and this phosphorylation in turn enhances the function of the channel [196]. 

Interestingly, formation of this tri-molecular complex is not ubiquitously observed in the brain. In the hippocampus, RACK1 is localized at the plasma membrane, where it scaffolds Fyn to the NMDAR. However, in the cortex, RACK1 is found mainly in the cell soma, and therefore it is not capable of compartmentalizing Fyn with the NMDAR. This specific compartmentalization of Fyn to the NMDAR complex *via* RACK1 may play a role in the actions of ethanol on the channel. In the hippocampus, upon ethanol treatment RACK1 is released from the NMDAR complex in a mechanism that depends on the activation of the cAMP/PKA signaling cascade. The dissociation of the tri-molecular complex enables Fyn to phosphorylate NR2B. These changes lead to phosphorylation-dependent enhancement of NMDAR channel activity during exposure to ethanol, leading to the development of acute desensitization and to a rebound potentiation of the channel activity when ethanol is washed out. However, in the cortex, because Fyn is not compartmentalized near NR2B, ethanol exposure does not result in changes in the phosphorylation state of the subunit [77, 197] and only the inhibition of channel activity but not the rebound potentiation following ethanol washout or the development of acute desensitization could be observed [197]. Together, these results suggest that in brain regions where the phosphorylation state of NR2B is regulated by the compartmentalization of Fyn *via* RACK1, ethanol’s actions on the NMDAR channel are the outcome of two opposing activities: 1) an increase in activity due to the release of RACK1 and the phosphorylation of NR2B by Fyn and 2) a decrease in activity due to the direct inhibitory activity of ethanol on the NMDAR as previously documented [102]. In addition, these results suggest a mechanism by which ethanol alters the phosphorylation state of a specific NMDAR subunit. This specificity of ethanol activity on the NR2B subunit in some brain regions but not in others is likely to contribute to the behavioral effects of ethanol, such as acute sensitivity to the hypnotic effects of ethanol, that are likely to be mediated *via* hippocampal Fyn and NR2B [115, 198].

## SUMMARY 

There is convincing preclinical evidence suggesting that adaptive processes taking place in the central nervous system in response to chronic ethanol administration contribute to the development of tolerance, dependence and withdrawal symptoms. Since NMDA receptors are among the highest-affinity targets for ethanol’s inhibition in the brain [57, 86], one of the most important adaptive changes may be the altered expression of these receptors. Up-regulation of NMDARs will result in the reduction of ethanol’s effect (tolerance), and accompanying neurochemical changes will lead to complex behavioral symptoms called ethanol dependence. Furthermore, the altered balance between excitatory and inhibitory processes will manifest in withdrawal symptoms when alcohol intake is ceased. The cellular events underlying the enhanced functioning of NMDARs in consequence of prolonged ethanol exposure include changes in the regulation of subunit expression, localization, post-translational modifications and interactions with other receptors or regulatory proteins. Better understanding of these processes can expand our knowledge on ethanol’s action that may establish new strategies for the treatment of alcohol-related diseases.

Our aim with this review was to summarize and give a complex view about all these events supposedly playing a regulatory role in the up-regulation of NMDAR functions in consequence of chronic ethanol exposure. In response to chronic blockade of NMDA receptors due to sustained ethanol administration, agonist and co-agonist induced NMDA receptor activation was shown to be enhanced. The augmented agonist activity is mostly accompanied with an increase in mRNA as well as protein levels of the NR1 and certain NR2, especially the NR2A and NR2B subunits [176, 88, 126]. The molecular mechanisms underlying these changes may consist of certain events in regulation of gene expression such as demethylation of the NR2B gene, enhanced NR2B promoter activity or down-regulation of a repressor (NRSF) of NR2B expression upon ethanol exposure. Regulatory steps occurring at the level of translation e.g. alterations in the half-life of NR1 mRNAs supposedly due to RNAi (microRNA) mechanisms may also contribute to these changes in subunit expression. Other regulatory events influencing the activity of NMDARs are the post-translational modifications, especially the phosphorylation state of the receptor. Activity of several kinases (Src, Fyn, PKA, PKC, CaM kinase II, CDK5) and phosphatases (PP1, PP2B) on NMDAR subunit phosphorylation was shown to be affected by ethanol leading to altered ethanol sensitivity and/or functional activity of NMDARs. As main structural components of the postsynaptic density, several scaffolding proteins like PSD-95, RACK1 and Yotiao also modulates the phosphorylation state of the NMDARs. These proteins and other cytosceletal elements regulating subunit assembly and distribution of NMDARs within different cell compartments, like α-actinin-2 and spectrin, as well as their synaptic/non-synaptic localization may influence glutamate sensitivity, therefore activity of certain neurons. There are some observations describing altered functions of these components regulating NMDAR functions upon ethanol exposure. Finally, ethanol also influences the co-operation of NMDARs with other neurotransmitter receptors such as mGluRs, dopamine, purine and adenosine receptors. Therefore, ethanol acting on the inter-receptor communication as well as on the above mentioned regulatory processes may contribute to its behavioral effects and to the development of alcohol tolerance, dependence and to the manifestation of withdrawal symptoms.
